# Engineering Rhizobial Bioinoculants: A Strategy to Improve Iron Nutrition

**DOI:** 10.1155/2013/315890

**Published:** 2013-11-06

**Authors:** S. J. Geetha, Sanket J. Joshi

**Affiliations:** Department of Biology, College of Science, Sultan Qaboos University, P.O. Box 36, Muscat 123, Oman

## Abstract

Under field conditions, inoculated rhizobial strains are at a survival disadvantage as compared to indigenous strains. In order to out-compete native rhizobia it is not only important to develop strong nodulation efficiency but also increase their competence in the soil and rhizosphere. Competitive survival of the inoculated strain may be improved by employing strain selection and by genetic engineering of superior nitrogen fixing strains. Iron sufficiency is an important factor determining the survival and nodulation by rhizobia in soil. Siderophores, a class of ferric specific ligands that are involved in receptor specific iron transport into bacteria, constitute an important part of iron acquisition systems in rhizobia and have been shown to play a role in symbiosis as well as in saprophytic survival. Soils predominantly have iron bound to hydroxamate siderophores, a pool that is largely unavailable to catecholate-utilizing rhizobia. Outer membrane receptors for uptake of ferric hydroxamates include FhuA and FegA which are specific for ferrichrome siderophore. Increase in nodule occupancy and enhanced plant growth of the *fegA* and *fhuA* expressing engineered bioinoculants rhizobial strain have been reported. Engineering rhizobia for developing effective bioinoculants with improved ability to utilize heterologous siderophores could provide them with better iron acquisition ability and consequently, rhizospheric stability.

## 1. Introduction

The best known and most exploited symbiotic N_2_-fixing bacteria are those belonging to the family Rhizobiaceae (Rhizobia) and include the following genera: *Rhizobium*, *Bradyrhizobium*, *Sinorhizobium*, *Azorhizobium*, *Mesorhizobium*, and *Allorhizobium* [1, 2]. Bacteria of these genera have the ability to infect the roots of leguminous plants, causing the formation of a new organ called nodule and establishing a nitrogen-fixing symbiosis. Rhizobium and its allies are Gram-negative bacteria that cause the development of root and also stem nodule on plant hosts, which the bacteria inhabit as nitrogen-fixing endosymbionts. There are sequence of events that are necessary for symbiotic nitrogen fixation which includes preinfection, root colonization, root adhesion, hair branching, hair curling, infection, nodule initiation, bacterial release, bacteroid development, nodule function, nitrogen fixation, complementary functions, and nodule persistence [3]. Under natural conditions, the rhizobial strains which quickly respond to the host signals, initiating the infection and nodulation quickly and efficiently are guaranteed to have a competitive advantage over the slower ones [4]. Rhizobial bacteroids fix atmospheric nitrogen in a form that the plants can utilize and in turn the bacteroids are supplied with an environment rich in carbon as an energy source. These bacteria infect legumes and are distributed globally [5]. The rhizobial N_2_ fixing ability varies greatly amongst the host plant species and bacterial strains [6, 7]. Thus, rhizobial-host compatibility must be taken into account while selecting the best strain as a bioinoculant. The additional qualities required for considering a rhizobial strain for practical applications as a bioinoculant are that it must efficiently improve the chances of nodulation and have a high N_2_-fixing rate.

In order to efficiently improve the chances of nodulation, rhizobia must compete successfully against a variety of soil inhabitants [8]. The definition of competition can be provided in number of ways but generally is considered to be a rivalry or a conflict between microbes for nutrition, space, or any other essential limiting factors. Thus, within the rhizosphere of a susceptible plant host the rhizobia has to compete for its nutrients with the indigenous soil microflora which consists of indigenous rhizobial species. Rhizobial bioinoculants fail to nodulate their host mainly because of highly competitive indigenous rhizobia capable of nodulating the same legume species. Competitiveness between two rhizobia is thus measured by the relative ability of the individual rhizobial strain to occupy the nodule [9]. These rhizobia may nodulate the host plant but fix little or no nitrogen, thus, reducing the nitrogen-fixing process. It can be suggested that the poor nodulation could be due to the inability of an inoculant strain to replace the successfully established population of rhizobia in soil. Thus, in order to increase the probability of successful nodulation by rhizobial inoculants, it is essential to first find out the reason for the observed failure and improving the same. 

From an ecological standpoint, the first step towards improving rhizobial inoculants must be to determine those aspects of the environment that can limit nodulation. There are reports from laboratory and field trials which state that there are abiotic and biotic factors like temperature, soil type, desiccation, pH, fertilizers, fungicides, resident protozoa, fungi, actinomycetes, rhizobacteria, and the nutrient supply (especially iron as it is limiting in soil) that may adversely affect nodulation of legumes by rhizobial bioinoculant strains [10]. 

There are two possible ways by which the effect of a desired bioinoculant could be improved (1) by modifying the environment to suit the bioinoculant strain and (2) to modify the rhizobial bioinoculant to suit the environment. The first approach is practically difficult under circumstances like modifying temperature, but the second alternative though costly and time consuming is practically possible. There may be two ways of achieving a modified rhizobial bioinoculant that suits the environment (1) by selecting a natural variant that is tolerant amongst all other rhizobial strains and (2) to genetically engineer a suitable inoculant strain. Thus, the objectives of our review was to consider the limitations that are imposed by the lack of iron as nutrition to rhizobial bioinoculant strains and to focus on ways to determine possible avenues for improving the competitive survivability of rhizobial bioinoculant strain by engineering the iron uptake machinery. 

## 2. Competitive Survival in Soils 

### 2.1. General Information from Various Bacteria

The rhizosphere of legume contains a large and heterogeneous community of bacteria. Interaction of these rhizosphere bacteria between each other and with other rhizobia are complex. Li and Alexander [11] observed an inhibition of the colonization and nodulation of alfalfa roots by *Rhizobium meliloti* in the presence of *Enterobacter aerogens*, *Pseudomonas marginalis*, *Acinetobacter* sp., and *Klebsiella pneumoniae*, whereas *Bradyrhizobium *sp. or *Micrococcus luteus *did not exert such effect on *Rhizobium meliloti*-alfalfa interaction. A correlation could be drawn between the growth rate of the rhizospheric bacteria and their effect on *Rhizobium meliloti*. The bacterial species having a faster growth rate than *Rhizobium meliloti* were acting as the competitors. The bacteria growing faster were preventing rhizobia from obtaining a large part of the excreted carbon needed to support cell growth. These effects may be caused due to the production of antibiotic compounds, siderophores, or plant growth hormones. The enhanced nodule occupancy by *Bradyrhizobium japonicum* USDA 110 in the presence of fluorescent *Pseudomonas* sp. was attributed to the iron availability and its influence on siderophore production by the *Bradyrhizobium japonicum* strains [12]. Another mechanism used by rhizobia to compete with rhizospheric bacteria, especially rhizobia noduling the same host, is by the production of bacteriocins [13]. 

#### 2.1.1. Competition for Iron in Rhizosphere

In natural environments there are always competitive interactions among the microbial inhabitants with their neighbors for the available space and resources [14]. Nutritional resources are the focal point of microbial competition and the success of an organism depends upon how efficiently it can acquire limiting essential nutrients, iron being one of them. 

In soils, rhizobia must not only be competent at solubilizing iron but also develop strategies to compete for this nutrient with other organisms present in the rhizosphere [15]. To meet iron requirements for growth, most microorganisms have developed specialized high affinity iron uptake system for scavenging and transporting ferric iron [16]. This includes machinery for the synthesis and secretion of low molecular weight compounds called siderophores which bind ferric iron with high affinity [17, 18] and several membrane bound and periplasmic proteins that function together in the uptake of the ferricsiderophore complex [19]. In addition to utilization of their own siderophore, *S. meliloti* and *B. japonicum* can also cross-utilize iron complex to siderophores made by other species (heterologous siderophore) [20, 21]. Most notable among the heterologous siderophores utilized by these rhizobia is ferrichrome, a prototypical hydroxamate type siderophore secreted by several fungi, including *Ustilago sphaerogena *[22] and *Ustilago maydis* [23]. Ability to utilize iron bound to ferrichrome is important from an ecological perspective since natural soils have significant concentrations of this siderophore [24, 25]. 

To gain an understanding of the iron distribution on a small scale on plants, Joyner and Lindow [26] developed a whole-cell biosensor to detect and measure bioavailable iron at the level of single bacterial cells. Ferric iron availability to cells of *Pseudomonas syringae* was assessed by quantifying the fluorescence intensity of single cells harboring a plasmid-borne transcriptional fusion of an iron-regulated promoter from a locus encoding a membrane receptor for a pyoverdine. Their results indicated that there is substantial heterogeneity of iron bioavailability to cells of *P. syringae* on plants, with only a small but significant subset of cells experiencing low iron availability. It is believed that chemical reactions and microbial respiration [27] can result in fluctuations in pH which in turn could affect the solubility of iron oxides, ultimately affecting the bioavailability of this element locally [28]. These results support models of competition for limiting iron resources among microbes on plants in that at least some of the cells in population encounter very low iron availability; thus, competition for this nutrient is an important aspect of rhizosphere ecology.

Competition for iron can be regarded as occurring in two stages either competition between excreted siderophores for the metal or competition between microorganisms for the Fe-siderophore complexes that are available in soil. The former is controlled by proton dissociation and formation constants of each siderophore, kinetics of exchange, and their concentration, while the latter is governed by the existence of an uptake mechanism for, and its affinity to, the Fe complex [29]. Fluorescent pseudomonads can suppress various soil borne plant diseases due to the production of siderophores and their densities in the rhizosphere [30, 31]. Pyoverdines are the major siderophores produced by fluorescent pseudomonads. They have very high affinity for Fe^3+^ with a stability constant of about 10^32^ [32] than the siderophores produced by other microorganisms that are deleterious to plant growth [33] and their membrane receptors are usually very specific [34]. These features enable the fluorescent pseudomonads to compete efficiently with the soil borne microflora. Pyoverdine-mediated iron competition has been shown to play a determinative role in the microbial antagonism performed by biocontrol strains against some pathogens.

**Figure 1:**
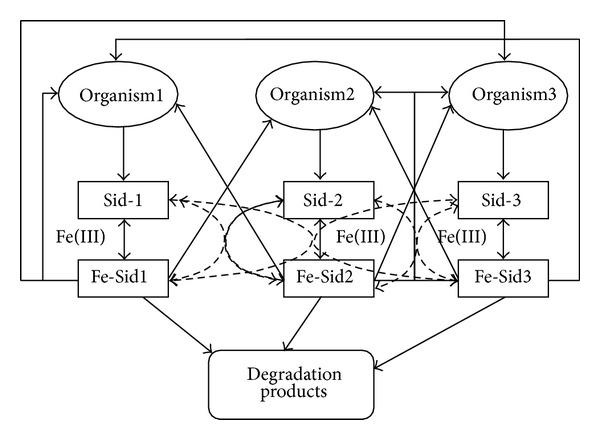
Model of siderophore mediated interactions between three organisms. The thick lines indicate transport by one organism of the Fe-Sid produced by the other organism. The dashed lines represent ligand exchange by which one Sid displaces another Sid chelating and Fe^3+^ iron. Degradation pathways, including biological and chemical mechanisms, are represented by the thin black arrowed lines.

As shown in Figure 1, siderophores may serve as Fe sources or as Fe competitors for organisms, depending on the ability of the organisms to acquire Fe from the stable Fe-siderophore complex. The siderophore mediated interactions as depicted in Figure 1 are for three organisms but may become much more complicated when more species are present.

Based on this concept it is obvious that if plant growth promoting (PGP) organisms (free-living or symbiotic) used as bioinoculant produce siderophore of significant high affinity, it will not only succeed to establish itself but will also be able to inhibit the growth of pathogenic organisms and hence can act as a biocontrol agent [35, 36]. 

## 3. Specific Information about Rhizobia

### 3.1. Rhizobia Competition for Iron: Affecting Its Rhizospheric Colonization

To induce nodulation on their leguminous hosts, the members of the genus *Rhizobium* probably must grow in the rhizosphere as well as at the site on the root hair where invasion of the plant begins. In these environments, the rhizobia are probably growing at the expense of organic products excreted by the roots, but these bacteria must compete for the essential nutrients (like iron) with other heterotrophic inhabitants of the rhizosphere. Many rhizobial strains have the potential to increase plant growth and yields but are poor at nodulation due to weak rhizospheric colonization. Many scientific studies have been performed that show the role of competition among strains of *Rhizobium *sp. for nodule sites and the impact of such intraspecific competition on nodulation. These works dealt with the behavior of mixed strains of nodule bacteria towards each other and towards their legume host, where they showed that when two strains of nodule bacteria were present in the surroundings of their host's root system, active competition between them caused the strain having a higher initial growth rate to overpower the other strain outside the plant. This dominant strain was then responsible for nearly all the nodules produced in the plant. Siderophore production and cross-utilization is a major microbial mechanism of interspecies competition; many bacterial species have the ability to utilize heterologous siderophores, sequestering iron away from the siderophore producer. Difference in iron binding affinities of siderophores, produced by different species can also mediate competition [37]. Several investigators have demonstrated that individual isolates of bacteria or fungi decrease the nodulation of aseptically grown white clovers by *R. trifolii* [38] or the rate and extent of nodulation of aseptically grown alfalfa by *R. meliloti* [39]. In these studies, it was shown that the organisms causing the suppression did not produce toxins against *R. trifolii* in culture, indicating that the suppression of nodule formation resulted from competition with the root nodule bacteria. 

There is little evidence that iron deficient soils affect the numbers of root nodule bacteria. In iron stressed soils, the proportion of siderophore-producing strains appear to increase. In alkaline soils of the midwestern United States *B. japonicum *serotype 135, a siderophore producer, has been shown to dominate over serotype 123, a siderophore nonproducer [40]. Similarly, 54% of *S. meliloti* strains isolated from alfalfa nodules from alkaline soil were siderophore producers, whereas from only 18% siderophore producing strains were obtained from an acidic soil [41]. These reports do suggest the possible advantage of siderophore production in iron deficient soil in the increased survivability and number of the producing strain in the rhizosphere. 

#### 3.1.1. Heterologous Siderophore Utilization: A Sound Strategy to Outcompete Other Rhizospheric Microflora

Complexation of Fe(III) by soil anions as well as competition for Fe(III) with other soil microorganisms are obstacles to iron acquisition commonly faced by *Rhizobium* sp. and all soil microflora. Rhizobia must be able to persist in the soil in the absence of their host plants and compete for iron, the availability of which is limited by its insolubility; this is one of the factors in determining how successful rhizobia are in maintaining themselves in the rhizosphere [42]. The rhizobial high affinity iron uptake systems may be of use in the competition among soil microbes for access to available iron and may enhance the survival of the free-living forms. Evidence has been presented to indicate that soil competition among rhizosphere pseudomonads may occur at the level of the uptake system of the ferric-complex specific for the individual *Pseudomonas *siderophore [43]. It has been shown that *R. meliloti* DM4 excretes and utilizes the siderophore rhizobactin, whereas several other wild-type *R. meliloti *strains do not [20]; thus, it is possible that a comparable competition might also occur among rhizobial strains. 

Certain rhizobia have also been shown to utilize iron bound to heterologous siderophores.* Bradyrhizobium japonicum* USDA110 and 61A152 can utilize ferrichrome (made by numerous fungi), rhodotorulate (made by yeast), pseudobactin (made by pseudomonads), and so forth [21], and these strains are successfully used as commercial bioinoculant for soybeans. *R. meliloti *DM4 could utilize the hydroxamate type siderophores, ferrichrome, and ferrioxamine B (made by actinomyces) in addition to its own siderophore rhizobactin [20]. Khan et al. [37] reported that a the growth of the strain that showed higher siderophore cross-utilization got stimulated in the presence of exogenously added siderophores in comparison to a low cross-utilizing strain which did not show such effects under similar conditions. Therefore, a rhizobial strain having the ability to utilize siderophore(s) produced by another similar rhizobial strain(s) (homologous siderophore) or from a nonrhizobial strain (heterologous siderophore) should also have a competitive advantage in the rhizosphere. It could be therefore said that possession of uptake system for the siderophore produced by majority organisms in the soil and hence predominantly present in the soil can have positive implications for growth and survival of the possessing organism. 

## 4. Improving Competitive Survival 

### 4.1. Strain Selection and Inoculant Preparation

Legumes have the most important role in agricultural productivity. Pulse and grain legumes are grown in the agricultural land in a rotation so as to fix atmospheric nitrogen into the soil because they form nitrogen fixing symbiosomes with soil inhabiting root nodule bacteria [44]. The successful use of legumes in agriculture will be dependent upon appropriate formation of effective symbioses with root nodule bacteria. An essential component for increasing the use of legumes is the integration of plant breeding and cultivar development with appropriate research leading to the selection of elite strains of root nodule bacteria. In addition research in these areas would increase benefits to the farmers through advances in the application of appropriate inoculation technologies to deliver the new strains of bacteria to the soil with enhanced survival abilities [8, 45]. A key feature of the symbiotic relationship between root nodule bacteria and legumes is the very high degree of specificity shown for effective nodulation of a particular host legume by a strain/of root nodule bacteria. This specificity operates at both the nodulation and nitrogen fixation levels of the symbiosis and is a function of the exchange of specific chemical signals between the two partners [46, 47]. The specificity is controlled by the fact that the chemicals can only be produced if the organisms contain the information for the synthesis of the chemicals on their genes. Thus, the development and function of the symbiosis between legumes and their associated root nodule bacteria is controlled at a molecular level, and the development of effective nitrogen-fixing symbioses is conditional on both partners containing appropriate sets of genes [47–49]. Three general strategies are possible when a new legume is introduced to new lands [50]. One is that the uninoculated legume may form abundant effective nodules indicating the presence in the soil of a large population of effective root nodule bacteria. In this case a plant breeding program to select a genotype able to nodulate effectively with the indigenous root nodule bacteria would be the simplest approach for successful introduction of the legume. Secondly, there is the less frequently encountered situation where no nodules are formed on uninoculated legumes. This result indicates there is very little or in some cases no background population of root nodule bacteria in the soil that are able to nodulate the particular host legume. More often, uninoculated legumes form variably effective nodules indicating the presence of a population of ineffective or variably effective root nodule bacteria. In the latter two scenarios the clear solution to ensure success with the legume crop is to select an effective, competitive inoculant strain of root nodule bacteria adapted to the soil conditions with the following desired characteristic: like effective nitrogen-fixation with the intended host species, stress tolerance, competitive ability against the indigenous strains, genetic stability, and satisfactory growth and survival during procedures for manufacture of inoculum [51]. 

### 4.2. Genetic Manipulations

The successful performance of rhizobial inoculant strains as we know depends upon their capability to out compete the indigenous soil bacteria, survive and propagate, and enter into effective symbiosis with the host plant. The strains which fail to survive under soil conditions are most of the time ineffective in enhancing legume productivity because vast majority of nodules formed are not by the inoculated strain but by indigenous rhizobia in the soil [52, 53]. Thus, construction of genetically engineered inoculum strains of *Rhizobium *with an increased ability to survive under soil conditions and hence compete for nodule occupancy was considered an amicable approach to address this problem. Most of the rhizobial biofertilizer strains are poor rhizospheric colonizers due to their inability to compete with the indigenous soil microflora for nutrients; iron being one of them because of the iron rich enzymes involved in nitrogen fixation [54]. Utilization of foreign siderophores is considered to be an important mechanism to attain iron sufficiency. Pseudomonads which are known for their rhizospheric stability have diverse iron uptake systems and multiple receptor genes have been detected in their genomes [55, 56]. From the above facts it could be suggested that by increasing the number of outer membrane siderophore receptors rhizobial strains could be made more efficient with respect to iron acquisition, and hence colonizing the rhizosphere. Most of the hydroxamate siderophores present in soil are of the ferrichrome-type. Because ferrichrome is synthesized by a variety of soil fungi, it is likely iron source in the rhizosphere where hydroxamate concentrations have been estimated to be as high as 10 *μ*M [57] and ferrichrome is found in nanomolar concentrations, as estimated by physicochemical [58] as well as bioassay methods [25]. As majority of soil bacteria are good utilizers of iron bound to hydroxamates [29], thus, the rhizobia isolates could be at a competitive disadvantage when residing free in soils. It is therefore pertinent to engineer these strains with a ferrichrome receptor to increase their iron acquisition property and hence survival. 

Thus, cloning of the ferrichrome receptor genein rhizobial bioinoculant strains and understanding the effect of ferrichrome utilization on rhizobial growth and survivability under conditions, wherein ferrichrome was made available by other producer species, were achieved by heterologously expressing the *E. coli fhuA* gene in *C. cajan* rhizobia. The *fhuA *was engineered under the control of the *lac *promoter and its expression was first confirmed in *E. coli* by the rescue of the phenotype of Δ*fhuA* mutant and was subsequently introduced into the rhizobial strains and its expression monitored. The expression of *E. coli fhuA* in rhizobial strains imparted them the associated phenotypes, namely, the ability to utilize iron complexed with ferrichrome and sensitivity to albomycin [59]. 

Several studies have shown that the utilization of heterologous siderophores by genetically introducing the receptor gene provides growth advantage to the bacteria. Brickman and Armstrong [60] found that the incorporation of alcaligin receptor gene alone could confer upon a siderophore deficient strain of *P. aeruginosa* the ability to utilize ferric alcaligin. Raaijmakers et al. [31] introduced the siderophore receptor for ferric pseudobactin 358 into *P. fluorescens* WCS374, resulting in a strain which was more competitive than the WCS374 parental strain for colonization of the radish rhizosphere. Benson et al. [61] showed that in addition to the FegA receptor required to form functional symbiosis, FegB was also necessary for *B. japonicum* to utilize ferrichrome. These observations reinstate the belief that among all the ligand-protein interactions of members of the bacterial iron-acquisition system, the binding of ferrisiderophores to the outer membrane receptor proteins is the most specific. For instance, in *E. coli* separate outer membrane receptors transport ferric iron bound to aerobactin, ferrichrome, rhodotorulic acid, and ferrioxamine, yet they all use a common set of periplasmic and inner membrane components. Genome sequences of bacteria contain numerous putative ferrisiderophore receptor genes [62, 63] but do not contain equivalent copies of the genes for periplasmic and cytoplasmic membrane-bound proteins. All the studies reported here favor the hypothesis that introduction of genetically engineered receptor genes helps in providing growth advantage to the Rhizobium species.

## 5. Engineering Iron Nutrition in Rhizobia 

### 5.1. Importance of Iron in Nodulation, Survival—General Findings

Although iron is the fourth most abundant element in the Earth's crust, it is essentially unavailable in aerobic environments at biological pH, as it tends to form insoluble Fe^3+^ oxyhydroxide [64]. To combat iron deficiency, different organisms produce different types of siderophores. In nature the ability to utilize various sources of chelated Fe appears to be of much importance and that is the reason why microorganisms often employ more than one high affinity Fe transport system for acquiring iron. This broad transport capability may be achieved by two means: either like the *E. coli* model which involves many specific siderophore transporters for uptake of ferrisiderophores that it may encounter or like the *Streptomyces* model which involves a nonspecific receptor/transporter for its own siderophore and other siderophores that has similar coordination of iron but different structures [15].

#### 5.1.1. Rhizobial Siderophores

Iron acquisition by *Rhizobium* sp. is essential for nitrogen fixation by the *Rhizobium*-legume root nodule symbiosis. Root nodule bacteria, form a nitrogen-fixing symbiotic interaction along with their leguminous plant hosts and have a high demand for iron [42]. Diverse types of siderophores are produced by the different rhizobial genera, for example, *Rhizobium leguminosarum bv. viciae*, the symbiont of peas, lentils, vetches, and some beans, synthesizes a cyclic trihydroxamate type siderophore called vicibactin, whereas *Sinorhizobium meliloti *2011 under iron stress produces rhizobactin 1021 a dihydroxamate siderophore [65]. Catecholate siderophores are known to be produced by rhizobia from the cowpea group [66] and salicylic acid and dihydroxybenzoic acid are produced by *Rhizobium ciceri* isolated from chick pea nodules [67]; citrate as a siderophore is produced by *Bradyrhizobium japonicum* [68] and anthranilate is produced by *Rhizobium leguminosarum*. 

Under natural conditions, the ability to produce siderophores has been demonstrated to confer a selective advantage to the producer organism [69]. It has also been speculated that besides capturing iron quotas necessary for growth, siderophores are also a type of iron scavengers because they can mobilize iron from weaker ferricsiderophore complexes from other species and thus are mediators of competitive interaction among organisms.

Microorganisms which themselves do not synthesize a particular type of siderophore may yet be proficient at the uptake of the iron-bound siderophore complex that they do not produce. The ability to cross-utilize heterologous siderophores may be accounted for by the presence of multiple type of siderophore receptors that are expressed for the uptake of different types of siderophores [62] or use of a low specificity (broad range) system that recognizes more than one type of siderophores [15]. Certain rhizobia have also been shown to utilize iron bound to heterologous siderophores. *Rhizobium meliloti *DM4 can utilize the hydroxamate type siderophores, ferrichrome (made by numerous fungi), and ferrioxamine B (made by actinomyces) in addition to its own siderophore rhizobactin [20]. *Bradyrhizobium japonicum* is able to utilize Fe^3+^ bound to two fungal siderophores rhodotorulate and ferrichrome [21]. 

 In natural environments such as the rhizosphere, the capacity to utilize heterologous siderophores produced by other members of the rhizosphere microflora is a positive fitness factor [31, 70]. Heterologous siderophores or its producer organism may bring about a variety of responses on other target bacterial species that are present within the same niche. Growth of some species is inhibited and this has been attributed to be one of the mechanisms by which biocontrol agent's act in inhibiting the growth of pathogens in the rhizosphere [33, 71]. In certain cases, addition of heterologous siderophores results in a growth stimulatory effect [37, 72]. Presence of exogenous heterologous siderophores can induce the expression of Iron Regulated Outer Membrane Proteins (IROMPs) in target bacterial species [73] and may also positively stimulate the target organism to synthesize its native siderophore [74]. 

#### 5.1.2. Iron Metabolism in Rhizobia

Siderophore-mediated high-affinity iron transport system comprises of an elaborate machinery involving genes for biosynthesis of siderophores, low-molecular weight iron chelating compounds, and specific receptors for the uptake of the siderophore-iron complex [19]. Nitrogen-fixing rhizobia, living as endosymbionts in root nodules of legume host plants, have a high demand for iron because a number of “symbiotic” proteins contain iron or heme [75]. Despite the importance of iron in these organisms, iron metabolism is relatively poorly studied in most rhizobial species. The lack of competitiveness for nodulation can be attributed to low intracellular concentration of iron in rhizobia [76]. The *S*.* meliloti *mutants, unable to acquire iron under Fe-limited conditions showed deficiency in competitive nodule occupancy. In soils, iron may be largely bound to siderophores secreted by various soil inhabitants. It has been shown that the siderophore ferrichrome, made by several fungi, is present in large amounts in soil solutions [24]. Organisms that do not synthesize ferrichrome may still be able to utilize iron bound to it, if they possess receptor for this siderophore. Thus, siderophores not only scavenge iron but also mediate antagonistic effect on other strains by depriving them of iron. 

Most of the rhizobial strains fail to utilize ferrichrome and other related hydroxamate type siderophores and are at competitive disadvantage in iron sufficiency [59, 77]. Heterologous receptors like (*fhuA*) from *Escherichia coli* or (*fegA*) from a *B. japonicum* strain that are proficient at utilizing ferrichrome have been expressed in rhizobial strains nodulating *Cajanus cajan* [59, 78, 79] and ground nut [80] plants. Engineering rhizobial strains by incorporating genes for multiple iron-siderophore receptors potentially increased the suite of ferricsiderophores that they could utilize. Plant inoculation studies performed at our lab showed increased nodulation efficiency and nodule number per plant by the engineered strains over the parental strains (nonengineered) under natural soil conditions. The transformants clearly benefited the plant also in terms of shoot fresh weight and increased chlorophyll content. Rhizospheric colonization and survival was also increased with transformants as compared to their parent under both autoclaved and unautoclaved conditions. Thus, it was demonstrated that increased survival of the strains does improve their nodule occupancy on *C. cajan *which in turn had positive effects on plant growth [78]. Similarly *fegA*-bearing strain when inoculated in unautoclaved soils with indigenous rhizobia (that could nodulate) showed better competitive nodulation ability [79]. Whether the competitive advantage of ferrichrome receptor expressing strains is restricted at the level of saprophytic survival alone or is also important during nodulation is under investigation.

### 5.2. Iron Acquisition Systems: Rhizobial Machinery

#### 5.2.1. Ferrisiderophore Uptake Machinery

Siderophores have been designed by nature in such a way that they are of sufficient size to engage the six octahedrally directed valence bonds of Fe(III); thus, they exceed the free diffusion limit of the small water-filled pores in the outer membrane of enteric bacteria. This in turn was sufficed by nature by the evolution of specific receptors for the recognition and transport of the iron-laden form of the siderophore. A siderophore system is thus comprised of two principal parts: the ligand and an arrangement for its uptake and utilization. Both parts of system are mostly subject to a common regulatory device triggered by the intracellular concentration of iron. The ferricsiderophore (Sid-Fe^3+^) from the extracellular medium is recognized by the N-terminal portion of a ferrisiderophore receptor, which serves two functions. First, the ferrisiderophore receptor transports Sid-Fe^3+^ into the periplasm, which is further transported into the cytoplasm by an ABC transporter. Both transport and induction functions require energy transduction from the TonB–ExbB–ExbD complex located in the inner membrane. Sid-Fe^3+^-bound ferrisiderophore receptor is believed to interact with TonB via its TonB-box motif [81]. 

#### 5.2.2. Siderophore Uptake Machinery and Its Regulation in Rhizobia

Although siderophore uptake machinery of rhizobia is poorly characterized, it can be said that this group of microorganisms have similar system of siderophore mediated uptake machinery as other Gram-negative species. Relatively few reports describe the presence of Iron Regulated Outer Membrane Proteins (IROMPs) in rhizobia that are produced and bind to their specific siderophore iron complex [82]. Stevens et al. [83] identified some of the *fhu* genes of *R. leguminosarum* which are homologues of the hydroxamate siderophores uptake machinery in *E. coli*. One of them is FhuA that specifies the OM receptor for uptake of vicibactin and works in association with FhuCDB (inner membrane proteins). FhuC is the ABC-transporter ATPase, FhuB is the permease and FhuD is the periplasmic siderophore binding protein which brings the ferrivicibactin complex to the inner membrane machinery for its transport from the periplasm to the cytoplasm [84]. Pseudogene versions of *fhuA* have also been detected in several other strains of *R. leguminosarum* [85]. Another receptor is rhizobial ferrichrome OM receptor FegA of *B. japonicum* 61A152 is a hydroxamate-type siderophore receptor [86]. The *fegA* gene is organized in an operon with *fegB* which probably encodes an inner membrane protein. Mutant analysis revealed that both genes are required for utilization of the siderophore ferrichrome [61]. FegA has been shown to bind ferrichrome specifically and its homologs have been detected in other strains as well. LeVier et al. [86] compared the amino acid similarities and identities among rhizobial receptor FegA with other nonrhizobial related siderophore receptor proteins. They have shown that FegA is 53.7% similar to the *E. coli *Fe(III)-ferrichrome receptor FhuA and 48.3% similar to coprogen and rhodotorulic acid receptor FhuE. Like any other ferrisiderophore uptake machinery the rhizobial high affinity Fe-uptake system also requires other components other than the receptor for the transport of the ferrisiderophore-complex. RhtA is an OM receptor responsible for rhizobactin uptake in *Sinorhizobium meliloti*, where a specialized single permease RhtX is responsible for its transport from periplasm to cytoplasm [87]. Wexler et al. [88] showed that *tonB*-like gene (*tonB*_*Rl*_) is adjacent to an operon that specified an ABC transporter involved in heme uptake in *R. leguminosarum*. The two of the *tonB*_*Rl*_ mutants, J350 and J344, had large halos on CAS plates due to higher amounts of the siderophore vicibactin that got accumulated in the extracellular medium. This was probably because the *tonB*_*Rl*_ mutants failed to import vicibactin and thus indicated that *TonB*_*Rl*_ was required for the Fhu transport system to operate, as found in other bacterial siderophore uptake systems. Whereas mutant analysis and complementation tests showed that the TonB system was specific for heme uptake and was dispensable for siderophore uptake proposing the existence of a second TonB homologue functioning in the uptake of Fe-chelates in *Bradyrhizobium japonicum* [89]. Regulation of ferrisiderophore assimilation system in rhizobia is mediated by Fur regulation which is highly conserved among most bacterial species; it is also present in *Bradyrhizobium japonicum* where the Fur protein has been shown to regulate the *hemA* gene [90] and *Rhizobium leguminosarum* [91]. The *B. japonicum *Fur proteins also have a novel function in the iron-dependent gene expression [92]. Another member of the Fur family is Irr (iron response regulator), which occurs in rhizobia. Irr was first identified in *B. japonicum*, as a transcriptional repressor of *hemB*, which specifies *δ*-aminolaevulinic acid dehydratase, in the heme biosynthetic pathway [93]. Irr is restricted to a few *α*-proteobacteria including rhizobia, *Agrobacterium*, the animal pathogen *Brucella *and *Rhodopseudomonas palustris* a photosynthetic bacterium very closely related to *B. japonicum*. It is moderately repressed in Fe-replete conditions, and this was shown to be dependent on a protein Fur_Bj_ that was homologous to Fur even though the irr promoter region had no sequence similarity to the canonical *fur* boxes [94]. These observations suggest that rhizobial Fur differs from that of other bacteria including *E. coli*. Recently Yang et al. [95] had shown the control of the expression of iron transport genes and many other iron-regulated genes which are not directly involved in heme synthesis in *Bradyrhizobium japonicum* through Irr. The Irr was shown to have both a positive and negative effect on the gene expression. Their findings indicated that *B. japonicum *sensed iron via the status of heme biosynthesis in an Irr-dependent manner and thus regulated iron homeostasis and metabolism.

Regulation of iron responsive genes in *R. leguminosarum *and* S. meliloti *is not mediated by Fur but rather by the dissimilar RirA (rhizobial iron regulator) protein a member of the Rrf2 family is a newly found gene whose product has no sequence similarity to Fur but which has close homologues in other rhizobia, like *Agrobacterium *and* Brucella* [96]. All the above reports state that the regulation of Fe-responsive genes in rhizobia is not mediated by Fur alone. There is also positive regulation reported in rhizobia. In the heme uptake system of *B. japonicum* encoded by the gene cluster hmuVUT-hmuR-exbBD-tonB, transcription of the divergently oriented *hmuT* and *hmuR* genes was not only found to be induced by iron limitation but to also depend on a 21-bp promoter-upstream iron control element (ICE) which by deletion analysis was shown to be needed for positive control [97]. 

### 5.3. Importance of Iron Acquisition Systems in Nodulation: Siderophore/Transporter Mutants

#### 5.3.1. Importance of Iron Acquisition in Rhizobial-Legume Symbiosis

There is a high demand for iron in the symbiotic interaction between legumes and rhizobia, although the mechanism by which it is provided in plant root nodules is not well understood. Recently Stacey et al. [98] studied the functional genome analysis of legume nodulation. A nodulated legume has an increased need for iron compared to a nonnodulated plant [99] since this metal is a constituent of key proteins such as nitrogenase and leghaemoglobin. Nitrogenase is made up of two proteins; both are rich in iron and essential for activity. Non-heme iron electron transfer proteins such as ferredoxin and flavodoxin are essential in nitrogen fixation [100]. Iron deficiency in nodulated legumes is very common on alkaline soils, and it affects common agricultural crops as chick pea [101], French bean [102], and peanut [103]. O'Hara et al. [104] reported the importance of iron in the legume-rhizobia symbiosis, where iron deficiency limits nodule development but not initiation in peanuts inoculated with *Bradyrhizobium *species. Nodulation is also drastically curtailed in lupines under iron deficiency [105]. Specific siderophore producing microorganisms stimulated the nodulation, nitrogen fixation and plant growth of leguminous plants [106, 107]. One of the possible modes of growth promotion of nodulated legumes under field conditions is production of siderophores which control the proliferation of soil-borne pathogens or facilitate the uptake of iron from environment [108, 109]. However, taking into consideration that iron stressed plants show fewer bacteroids present in the nodules, decreased amount of leghemoglobin and lower specific nitrogenase activity, and that possession of the ability to produce siderophore significantly increases the efficiency of the differentiated bacterium to fix nitrogen and induce an increase in plant growth. It can be suggested that the difference in nodule development under iron deficient conditions may be due to varying abilities of different strains of root nodule bacteria to acquire iron for nodule initiation and development.

*Rhizobium leguminosarum* 116, an ineffective mutant strain was shown to form white ineffective nodules on peas and had an apparent defect in iron acquisition [110]. Also the nitrogenase activity of plants inoculated with wild-type *R. meliloti *1021 that produced rhizobactin 1021 and rhizobactin 1021 mutants over a 70-day period showed a significant increase in nitrogenase activity and total plant dry weight in the wild type strain as compared to the rhizobactin 1021 mutant [111]; even the efficiency in nitrogen fixation increased when the plants were inoculated with the wild type strain compared to plants inoculated with rhizobactin 1021 mutants [112]. These studies suggest that rhizobactin 1021 contributes to the efficiency of nitrogen fixation under certain conditions of plant growth. Other studies have shown conflicting results: in some cases, rhizobial mutants defective in siderophore synthesis fix N_2_ normally [113] but in others Sid^−^ mutants fail to fix N_2_ symbiotically [114]. In a later study it was shown that, in iron-rich medium, the regulation of rhizobactin synthesis is a factor in efficient nitrogen fixation [115]. With the aim of elucidating the implication of iron and iron transport mechanisms in the three-partner interaction rhizobia- legume-soil, Fabiano et al. [116] evaluated the ability of rhizobia to express high-affinity iron transport systems and showed that iron acquisition systems are widespread in Uruguayan isolates. Studies with the native strain *S. meliloti *242 demonstrated that the siderophore-mediated iron uptake systems are not essential for an efficient biological nitrogen fixation but they are involved in early steps of nodulation and in rhizobial competitiveness [117]. Besides mutants defective in siderophore biosynthesis, those defective in other aspects of iron uptake and regulation also have been shown to give similar confusing findings. A *fur*-null mutant of *B. japonicum*, under derepressed state for iron uptake, is shown to form effective symbiosis [90], whereas a manganese-resistant *fur* mutant strain was unable to form functional, nitrogen-fixing nodules on soybean, mung bean, or cowpea, suggesting possible roles for a Fur-regulated protein or proteins in the symbiosis at least under some circumstances [118]. Yeoman et al. [85] isolated an *fhuA* mutant *Rhizobium leguminosarum*. The mutant was shown to be defective in iron uptake and accumulated the siderophore vicibactin but did not detectably affect symbiotic N_2_ fixation on peas. Also *fhuA::gus* fusion was expressed by bacteria in the meristematic zone of pea nodules but not in mature bacteroids suggesting that vicibactin is not used for iron uptake in *R*. *leguminosarum *bacteroids. Experiments show that both *fegA *and *fegB *are required for the utilization of ferrichrome, since the *fegB *mutant were found to form a normal symbiosis and fegAB mutant had a dramatic phenotype in planta leading to the speculation that it might be due to the inability of the mutant to transport ferrichrome. This symbiotic defect suggested that the *fegAB* operon was serving a different function in planta, possibly thought to be involved in the signaling between the rhizobia and the host plant [61]. It is generally believed that siderophore biosynthesis or transport genes are not expressed when nitrogenase genes are actively expressed. How the supply of iron is provided to form nitrogenase, at a stage in nodulation when it is highly active, remains to be resolved. It may be possible that iron nutrition of the bacteroids in the nodules is through ferrous form of iron which becomes available to the bacteroids through the reductases present on the peribacteroid membrane [119]. Recently while studying the expression of 200 genes of *S. meliloti* upon induction with plant symbiotic elicitor luteolin during symbiosis it was found that three genes related to iron metabolism were induced by luteolin and also in nodules. One of them coded for a probable siderophore, sitA for an iron transporter and another was homologous with a gene of the *tonB-hmu* cluster of *R. leguminosarum *[120]. SitABC-transporter thus seems to be an important system for iron acquisition in planta.

### 5.4. Engineering Strategy

Various different strategies have been applied to improve the competitiveness of a bioinoculant in the plant environment. These are either by promoting rhizobial multiplication in the plant environment, by inhibiting the growth of competing microorganisms, or by interfering with some of the signals perceived by the competing microbes provided these signals control (at least in part) the expression of functions central to microbial fitness [121]. Because this is a triple interface (bacteria, plant, and soil) interaction, it is possible to modify one, two, or three of these factors to improve microbial colonization. An improvement of plant-microbe symbioses should involve the coordinated modifications in the partner's genotypes resulting in highly complementary combinations [122]. Genetic manipulation of the bacteria should take into account genes which can be used to increase competitivity. While much of the effort has been directed to understand genes whose deficiency leads to loss of competitivity, only few studies deal with genes whose overexpression can improve competitivity. Successful strategies in this regard are as follows: (1) construction of a chimeric Nif HDK operon under the strong NifHc promoter and expression in PHB negative mutants of *R. etli* [123], (2) construction of an acid-tolerant *R. leguminosarum bv. trifolii* strain [124], (3) expression of ACC deaminase gene in *S. meliloti* [125], (4) overexpression of *putA* [126], (5) overexpression of trehalose 6-phosphate synthase [127], (6) overexpression of *rosR* and *pssR* [128], (7) heterologous expression of ferrichrome siderophore receptor gene *fegA* and *fhuA *[78–80], and (8) overproduction of the adhesion rap1 [129]. Of these, the strategy of Peralta et al. [123] explained that *R. etli* strains with the chimeric nitrogenase construct assayed in greenhouse experiments had increased nitrogenase activity (58% on average), plant weight (32% on average), N content in plants (15% at 32 days after inoculation), and most importantly higher seed yield (36% on average), higher N content (25%), and higher N yield (72% on average) in seeds. Additionally, expression of the chimeric *nifHDK* operon in a PHB-negative *R. etli* strain produced an additive effect in enhancing symbiosis. Probably, this is the first report of increased seed yield and nutritional content in the common bean, obtained by using only the genetic material already present in Rhizobium. Mongiardini et al. [129] on the other hand, investigated the influence of adhesins on competitiveness of *R. leguminosarum bv. trifolii* using clover as test plants. In this report, the *R. leguminosarum bv. trifolii *adhesion protein RapA1 was overproduced from a pHC60-derived plasmid and expressed in R200 strain. When an overproducing strain and a control-carrying empty vector were coinoculated on clover plants, a positive effect of RapA1 on competition for nodule occupation was observed suggesting that optimization of RapA1 expression may be considered while improving the rhizobial competitiveness. Ability to cross-utilize heterologous siderophores is another trait that can be incorporated into bioinoculants to further improve their competitiveness. The release of genetically improved strains is often restricted due to lack of regulation and proper guidelines of its release besides its potential ecological effects, as perceived by the public. However, despite these perceptions, some recombinant rhizobial strains have been commercialized, such as *S. meliloti* strain RMBPC-2, which was approved by the US Environmental Protection Agency in 1997. This genetically engineered bacterium contained additional copies of *nifA* and *dctABD* to increase nitrogen fixation and when inoculated, enhanced the yield of alfalfa [130] under N-limiting conditions. Later on, inoculation of alfalfa seeds with any of the three recombinant strains of *S. meliloti* significantly increased overall plant biomass compared with inoculation with the wild-type strains over a 3-year period during which high proportion of nodules were occupied by the inoculum strains. However, the recombinant strains were found to be ineffective in soils where the indigenous rhizobial populations were more competitive [131]. 

#### 5.4.1. Bioinformatics Details about Presence/Absence of Genes in Different Rhizobia

Various techniques like mutations, deletion mapping, cloning vectors, and so forth. have facilitated the identification of genes associated with nitrogen fixation. Legume, nonlegume, and free living nitrogen fixers have a set of genes which are responsible for effective nodulation and nitrogen fixation. These are the *nod*, *nol*, *noe*, *nif*, *fix*, and some hydrogenase genes. The work on the genetics of nitrogen fixation was first started in *Klebsiella oxytoca* M5a1 and first ever detailed organization of *nif* genes were reported in this organism [132]. A number of studies have established that core *nif* genes like *nifH*,* nifD, nifK*,* nifY*,* nifB*,* nifQ*,* nifE*,* nifN*,* nifX*,* nifU*,* nifS*,* nifV*,* nifW*, and *nifZ* are essential for nitrogen fixation. On the basis of mutational studies the natures of different *nif* gene products were determined. Studies confirmed that *nif* HDK encodes nitrogenase while *nif* LA had regulatory functions. In cyanobacteria especially *Anabaena* sp. 7120 it was established that the organization of *nif* HDK differed from that of *Klebsiella pneumoniae. *Rao [133] reported that it was Johnston and his coworkers who discovered the presence of nodulation genes in a plasmid of *Rhizobium leguminosarum* and mutation of those genes rendered them useless. Like the rhizobia, *Azosprillium* includes a megaplasmid and sequences similar to *nod* genes [134] Later on studies ascertained that *nod*, *nol*, and *noe* genes produce nodulation signals [47, 135]. The interplay of different *nod *genes, triggering of the creation of root nodule, signaling cascades, and development of nodule meristem were reported by a number of researchers [136, 137]. Studies concerning nodule physiology and nodule functioning specified that the bacteria in bacteroids forms are surrounded in plant membranes resulting in the formation of symbiosomes [138]. *Frankia* houses a number of *nif* genes but researchers failed to spot *nod* genes in *Frankia* [139]. However, groundwork on *Frankia* genomes exposed some putative *nod*-like genes which did not emerge in organized clusters and failed to detect the *nodA* gene [140]. Rao [133] reported that *fix* genes were identified on the chromosomes as well as plasmids of *Rhizobium* and were transferable. These genes are irregular in free-living bacteria and assist in electron transfer to the nitrogenase. The amalgamation of the knowledge of plant physiology, biochemistry, genetics, and molecular biology gave idea about the understanding of the mechanism of nitrogen fixation in pregenomic era. Things changed with the accessibility of complete genome sequences of symbiotic as well as nonsymbiotic diazotrophs. Knowledge of the whole genome became the stepping stone in understanding the working principle of the bacterial cell. *Mesorhizobium loti* was the first sequence of a symbiotic bacterium and it was followed by *Sinorhizobium meliloti* [141]. The completion of the genomes of *Rhizobium leguminosarum bv. viciae* [142], *Rhizobium etli* [143], *Bradyrhizobium japonicum* starin USDA110 and USDA6^T^ [144, 145] *Frankia* strains [146], and sequences for a number of free-living diazotrophs spanning different habitats and ecological niches [140] bolstered nitrogen fixation research. The studies on the genomes exposed new evidences pertaining to evolution and structure, interactions between plants and microbes. The detection of a number of symbiotic genes associated with nitrogen fixation has strengthened functional genomic research. Tools like DNA macro- and microarrays have been applied for studying expression at transcription level in *Sinorhizobium* and are being applied in other rhizobia [141]. Competitive ability of the bioinoculants strain in comparison to the indigenous microflora is governed not only by its ability to fix nitrogen (governed by *nif* genes) but also by the genes involved in the iron uptake system as they play a key role in proper nodule development. Pseudomonads are known for their rhizospheric stability and one of the factors contributing to this is the presence of diverse iron uptake systems. About 32 putative siderophore receptors in *P. aeruginosa *[55, 56], 29 in *P. putida*, 27 in *P. fluorescens*, and 23 in *P. syringae* [62] are reported. Analysis of protein sequences of complete genomes of *Pseudomonas* species using HMMER profiles revealed the presence of 45 TonB dependent siderophore receptors in the genome of *P. fluorescens*, 31 in *P. putida*, and 36 in *P. aeruginosa*. In contrast, a complete genome wide search in a few members of rhizobiales revealed a visible scarcity of TonB dependent siderophore receptors: 3 were present in *R*. *etli*, 3 in *Mesorhizobium *sp. BNC1, 2 in *Mesorhizobium loti*, 2 in *Sinorhizobium meliloti*, and 8 in *Bradyrhizobium japonicum *[79] Relatively high number of TonB dependent receptors present in *Bradyrhizobium *sp. amongst rhizobiales [147] and this could be attributed for their high rhizospheric competence and hence their reported success as commercial biofertilizers for soybean crops. In* R. leguminosarum *genome, the iron-siderophore uptake machinery was present on plasmid pRL, different from other rhizobia, where the iron uptake machinery is present on the genome [148] clustered together as FhuABDC (GI: pRL100325, GI: pRL100326, GI: pRL100327, and GI: pRL100328). The other FhuA homologs are not found to be associated with inner membrane machinery, similar to FhuE (rhodotorulic acid and coprogen receptor) and IutA (aerobactin receptor) of *E. coli*, which are also found in isolation [53]. These receptors work in association with FhuBCD (ferrichrome system) suggesting that the transport of ferrisiderophores through the inner membrane is not as specific as that through the outer membrane. It is due to this reason that a notably less number of periplasmic and cytoplasmic membrane proteins are reported to be present in *Bradyrhizobium *sp. and *Pseudomonas *sp. against a relatively large number of outer membrane receptors reported [83]. These findings led us to hypothesize that increasing the repertoire of outer membrane siderophore receptors could make rhizobial isolates more efficient with respect to iron acquisition and hence colonization of the rhizosphere. 

#### 5.4.2. Overexpression of Receptor and Findings

The successful performance of rhizobial inoculant strains depends upon their capability to out-compete the indigenous soil bacteria, survive, propagate, and enter into effective symbiosis with the host plant. Biofertilizer strains which fail to survive under soil conditions are most of the time ineffective in enhancing legume productivity because vast majority of nodules formed are not by the inoculated strain but by indigenous rhizobia in the soil [53, 104]. *Rhizobium *sp. ST1 was inhibited slightly in the presence of ferrichrome under lab conditions and also under natural soil conditions, but the expression of *fhuA* genes into *Rhizobium* sp. ST1 increased its rhizospheric stability, which was evident by its increase in nodule occupancy on the *C. cajan* plants [59, 78, 149]. The *fegA* gene with its native promoter when subcloned and transferred into *Mesorhizobium *sp. GN25 and *Rhizobium* sp. ST1, imparted these strains the ability to utilize ferrichrome, and increased their rhizospheric competitive abilities [79]. Similar to the above findings, where the expression of only the outer membrane receptor is shown to impart siderophore utilization, Brickman and Armstrong [60] showed that the expression of *fauA *gene, encoding receptor for alcaligin siderophore, imparts alcaligin utilization to a *P. aeruginosa *strain deficient in alcaligin production [60]. These findings provide evidence that engineering rhizobial strains with ferrichrome utilization ability provides them with a competitive edge in an environment where Fe-ferrichrome is the only available source of iron. Whether siderophore production or uptake efficiency increases the nitrogen fixation ability of nodule bacteria is not yet known, but their survival in the absence of their host definitely depends upon this characteristic.

## 6. Conclusion 

Bacteria may become competitively successful only if armed with the appropriate tools of efficient substrate acquisition, resistance mechanisms, and competitive traits. Comparative genomics analyses have revealed the existence of competitivity genes that are conserved and many that are specific to each species. Signature-tagged mutant libraries constructed for *S. meliloti* and *M. loti* are an important and powerful resource for future functional genomics and such libraries of other strains may be useful in understanding and engineering rhizobia for better legume productivity in different agro-ecological regions of the world. Molecular biology together with screening genotypes may help to identify and concurrently develop more effective inoculants strains. Even though the use of genetically modified microbial inoculants in agriculture is controversial, future demands on agricultural produce, ever-growing populations, and decreasing cultivable lands require a regulated and lawful use of genetically engineered inoculants. A very careful assessment of genetic modification of rhizobia have to be made so that after release into soil it does not have any deleterious impact on indigenous microbial communities of agronomic importance. The competitive advantage conferred by genes involved in metabolic fitness and nutrient acquisition are perhaps relatively harmless in nature, whereas overexpression of genes coding for the synthesis of antibiotic-like molecules may affect other indigenous microbial species and may alter bacterial diversity. Recent interest in application of rhizobia to enhance growth of nonleguminous plants like rice, sugarcane, wheat, and maize either as associative symbionts or as endophytes extends the use of this group of microorganisms for plants other than legumes. Applying rhizobial inoculation technology to the nonleguminous plants, however, may cause competition problem to such plants. Therefore, the factors affecting establishment of inoculated rhizobia as endophytes need to be considered and competitive effect of native population should be addressed. Whether increasing competitiveness for nodulation also enhances endophytic competiveness could be explored.
